# Association of total pre-existing comorbidities with stroke risk: a large-scale community-based cohort study from China

**DOI:** 10.1186/s12889-021-12002-1

**Published:** 2021-10-21

**Authors:** Ya Zhang, Cuicui Wang, Dong Liu, Zhengyuan Zhou, Shujun Gu, Hui Zuo

**Affiliations:** 1 School of Public Health, Medical College of Soochow University, 199 Ren’ai Rd., Suzhou, 215123 China; 2 Jiangsu Key Laboratory of Preventive and Translational Medicine for Geriatric Diseases, Medical College of Soochow University, 199 Ren’ai Rd., Suzhou, 215123 China; 3 Changshu Center for Disease Control and Prevention, 6 Fuyang Rd., Suzhou, 215500 China

**Keywords:** Pre-existing comorbidity, Stroke, Risk, Cohort studies

## Abstract

**Background:**

Comorbidities, any other coexisting diseases in patients with a particular index disease, are known to increase the mortality of a stroke. However, the association of pre-existing comorbidities with stroke risk has not been fully studied.

**Methods:**

This study included 16,246 adults from a prospective community-based cohort with a baseline survey conducted in 2013 in China. Participants were followed up with hospitalization records and the Cause of Death Registry. The association of eight pre-existing comorbidities (coronary heart disease, hyperlipidemia, hypertension, diabetes, previous stroke, chronic obstructive pulmonary disease, nephropathy, and cancer) with stroke risk was analyzed using the Cox proportional hazard model in 2020.

**Results:**

At a median follow-up of 5.5 years, a total of 449 participants (206 men and 243 women) developed a stroke. Four pre-existing comorbidities (hypertension, congenital heart disease, previous stroke, and diabetes) were independently and positively associated with the risk for all types of stroke. The adjusted hazard ratios for participants with only 1 and ≥ 2 pre-existing comorbidities compared with those without pre-existing conditions were 1.96 (95% CI: 1.44, 2.67; *P* < 0.001) and 2.87 (95% CI; 2.09, 3.94; *P* < 0.001) for total stroke, respectively. Moreover, male and female participants with a combination of increased age and a higher number of pre-existing comorbidities experienced the greatest risk of stroke.

**Conclusions:**

The number of pre-existing comorbidities was independently associated with an increased risk of stroke. There was a synergic effect between increased age and a higher number of pre-existing comorbidities on stroke occurrence. Our novel findings emphasize the importance and potential application of pre-existing comorbidities as a risk indicator in stroke prevention.

**Supplementary Information:**

The online version contains supplementary material available at 10.1186/s12889-021-12002-1.

## Background

Stroke is ranked as the second leading cause of death and a major cause of disability in the world [1]. This phenomenon is likely caused by the accumulation of risk factors in aging populations [2]. Each year, about 795,000 people experience a new or recurrent stroke [2], leaving 26% of them with a disability in basic activities and 50% with reduced mobility due to hemiparesis [3]. Approximately 3 to 4% of total health care expenditures in Western countries are spent on stroke [4]. In China, stroke is currently the leading cause of death and one of the main causes of disability [5, 6], and therefore represents a heavy psychological and economic burden to families of patients and society.

Comorbidity refers to any other coexisting disease in patients with a particular index disease known to shorten the life span and increase mortality in patients [7, 8]. Currently studies of comorbidity mainly focus on the burden of disease [9–11], rather than pre-existing comorbidities as risk factors of disease, limiting the application of pre-existing comorbidities in public health and clinical treatment.

Stroke shares common risk factors with some chronic diseases [12] [e.g., coronary heart disease (CHD), hypertension, diabetes, and hyperlipidemia], such as age [5], sex [13], hypertension [14], smoking [15], metabolic syndrome [16], physical inactivity [17], and diet and nutrition [18]. With increasing age, the accumulation of these systemic risk factors may further increase the possibility of developing a stroke [5]. Previous studies showed that comorbidity was a strong prognostic factor for stroke mortality [19, 20]. Some psychiatric comorbidities have been shown to be associated with stroke risk [21–23]. However, existing studies on comorbidity and stroke mostly focused on a single comorbidity and its association with clinical endpoints such as survival rate, quality of life, and health care utilization. The impact of multiple pre-existing comorbidities on the risk of stroke in a general population remains inconclusive. Understanding the effect of pre-existing comorbidities on stroke risk would help us improve stroke prevention strategies. Therefore, in this study, we conducted a prospective analysis to reveal the relationship between eight pre-existing comorbidities (CHD, hyperlipidemia, hypertension, diabetes, previous stroke, chronic obstructive pulmonary disease (COPD), nephropathy, and cancer) and stroke risk in a Chinese community-based cohort.

## Methods

### Study population

This study was based on a community-based cohort with a baseline survey conducted in 2013 in Changshu, eastern China. The source population was mostly from participants in an earlier study on metabolic syndrome in 2008 [24, 25]. A multi-stage sampling method was used to recruit participants from Changshu, China, which is located in the south of Jiangsu province, one of the richest areas in China. Specifically, six towns or streets were randomly selected, and two village-level communities from each township or streets were then randomly sampled. All participants were sampled from locally registered residents in the communities mentioned above. After the removal of individuals with severe cancer, severe disability, or severe psychiatric disturbance, a total of 16,457 adults participated in the baseline survey. Of these, participants with missing data on health information at baseline (*n* = 211) were excluded, leaving 16,246 participants (6650 men and 9596 women) for the final analyses. All analyses were conducted in 2020.

### Measurements

Pre-existing comorbidities [CHD, hyperlipidemia, hypertension, diabetes, previous stroke, COPD, nephropathy, and cancer], together with family history, demographic characteristics, and lifestyle factors were measured with standard questionnaires administered by trained staff. Blood samples were collected in the morning after at least 8 h of overnight fasting and all biological and clinical parameters were assessed on the same day of physical examination. The levels of total cholesterol (TC), triglyceride (TG), and high-density lipoprotein cholesterol (HDL-C) were measured enzymatically using commercial reagents with an automatic biochemistry analyzer (Hitachi Inc., Tokyo, Japan). The levels of low-density lipoprotein cholesterol (LDL-C) were calculated by the Friedewald formula [26]. Fasting blood glucose was measured using an oxidase enzymatic method. Blood pressure was measured in the sitting position three times every 30 s with an electronic sphygmomanometer (Omron Hp1300, OMRON Corporation, China) by a trained observer after participants rested for 5 min. Height and weight were measured by standard methods, and the body mass index (BMI) was calculated as weight in kilograms divided by the square of height in meters.

### Definition of pre-existing comorbidity

Comorbid diseases were integrated with two widely used methods of comorbidity measurement—Charlson Comorbidity Index [27, 28] and Simplified Comorbidity Score [29]. A panel of eight essential comorbidities, including hypertension, CHD, previous stroke, diabetes, hyperlipidemia, COPD, cancer, and nephropathy was selected and characterized at baseline. Participants were considered to have hypertension if they reported a history of hypertension, used antihypertensive medication, or had a blood pressure ≥ 140/90 mmHg upon physical examination. Participants were considered to have diabetes if they reported a personal history of diabetes, used hypoglycemic drugs, or possessed a fasting blood glucose level of ≥7.0 mmol/L. Hyperlipidemia were defined based on self-reported disease history, the use of lipid-lowering treatment, or laboratory measurement (TC > 6.20 mmol/L, TG > 2.30 mmol/L, LDL-C > 4.10 mmol/L and HDL-C < 1.00 mmol/L) [30, 31]. Nephropathy was defined as self-reported nephropathy history, use of nephropathy treatment, or glomerular filtration rate < 60 mL/min. Information regarding CHD, previous stroke, cancer, COPD and nephropathy at baseline was based on the self-reported survey. CHD included a history of heart failure, angina pectoris, and/or myocardial infarction. Previous stroke included hemorrhage, ischemic stroke, subarachnoid hemorrhage, and any other undetermined types of stroke. Cancer included all types of malignant solid tumors. COPD was defined as any type of obstructive lung disease characterized by long-term breathing problems and poor airflow. The number of separate pre-existing comorbidity conditions for each participant was totaled. Accordingly, participants were classified into three groups, based on the total number of pre-existing comorbidities (0, 1, and ≥ 2).

### Follow-up and ascertainment of stroke

Follow-up began at the baseline and ended at the date of stroke occurrence, death, or December 31, 2018, whichever came first. To obtain accurate recording linkages, we followed up cases with hospital discharge diagnoses and the Cause of Death Registry. Follow-up was 100% complete, with a total of 87,897 person-years. Besides total stroke, we collected information on the type of stroke, ischemic or non-ischemic (including hemorrhagic stroke, subarachnoid hemorrhage, and unexplained types of stroke). The primary outcome was hospitalization or death attributed to stroke (total stroke: ICD-10 codes I60-I61, I63-I64; ischemic stroke: ICD-10 codes I63 except for I63.9). If > 1 stroke events occurred in a participant during the follow-up period, only the first event was considered.

### Statistical analysis

Baseline demographic characteristics are presented as median (interquartile ranges) for continuous variables and reported as percentages for categorical variables. Between-group differences were tested by the Chi-square tests for categorical and Kruskal-Wallis tests for continuous variables, respectively.

Cox proportional hazard analyses were conducted to evaluate each pre-existing comorbidity and risk of stroke, and results presented as hazard ratio (HR) and 95% confidence intervals (CI). We then calculated the associations between the number of pre-existing comorbidities (0, 1, and ≥ 2) and stroke risk. In the minimally adjusted models, we adjusted for sex and age (continuous). In the multivariable-adjusted models, we additionally adjusted for conventional risk factors including BMI, current smoking, and current drinking. In addition, we examined the potential joint effects of pre-existing comorbidity and age (< 60 years and ≥ 60 years) by the Cox models. We used the interaction term Relative Excess Risk due to Interaction (RERI_HR_ = HR_11_ – HR_10_ – HR_01_ + 1; RERI_HR_ > 1 represents a positive interaction) to evaluate the joint effect [32]. Sensitivity analyses were performed to determine the robustness of findings in the primary analysis, by repeating the analyses after excluding the first 1 year of follow-up.

Statistical analyses were conducted with R (version 4.0.3). All tests were two-sided, and a *P* value less than 0.05 was considered statistically significant.

## Results

### Characteristics of the study population

Among the 16,246 participants, a total of 449 (206 men and 243 women) developed stroke over a median follow-up of 5.5 years, of which 351 (78.2%) were ischemic. The most common pre-existing comorbidities were hypertension (prevalence 45.4%), followed by hyperlipidemia (prevalence 30.7%), and diabetes (prevalence 9.0%). Baseline characteristics of the participants stratified by number of comorbidities are shown in Table 1. Compared with participants without pre-existing comorbidities, those with a higher number of pre-existing comorbidities were more likely to be older, men, current drinkers and have a higher BMI (*P* < 0.001), whereas there was no difference in smoking status among the three groups.

**Table 1:** Baseline characteristics of the study population by total pre-existing comorbidities (*n* = 16,246)

		Total pre-existing comorbidities			*P* Value^2^
		0 (*n* = 6049)	1 (*n* = 6555)	≥ 2 (*n* = 3642)	
Age, years ± SD		50.22 ± 12.48	57.33 ± 12.55	60.23 ± 11.10	< 0.001
Follow-up time, years ± SD		5.48 ± 0.39	5.40 ± 0.65	5.32 ± 0.83	< 0.001
BMI^1^, means ± SD		22.47 ± 2.95	23.80 ± 3.26	24.94 ± 3.36	< 0.001
Gender	Female n (%)	3820 (63.2)	3756 (57.3)	2020 (55.5)	< 0.001
Current smokers n (%)		1385 (22.9)	1586 (24.2)	857 (23.5)	0.229
Current drinkers n (%)		879 (14.5)	1113 (17.0)	623 (17.1)	< 0.001
Comorbid conditions	Hypertension, n (%)	0	3969 (60.5)	3411 (93.7)	< 0.001
	CHD^1^, n (%)	0	21 (0.3)	123 (3.4)	< 0.001
	Hyperlipemia, n (%)	0	2220 (33.9)	2765 (75.9)	< 0.001
	Diabetes, n (%)	0	258 (3.9)	1201 (33.0)	< 0.001
	Previous Stroke, n (%)	0	5 (0.1)	158 (4.3)	< 0.001
	Cancer, n (%)	0	29 (0.4)	124 (3.4)	< 0.001
	COPD^1^, n (%)	0	8 (0.1)	33 (0.9)	< 0.001
	Nephropathy, n (%)	0	45 (0.7)	157 (4.3)	< 0.001

^1.^*BMI* body mass index, *CHD* coronary heart disease, *COPD* chronic obstructive pulmonary disease

^2.^*P* Values were determined by Chi-square tests for categorical and Kruskal-Wallis tests for continuous variables

### Pre-existing comorbidities and the risk of stroke

**Fig. 1:**
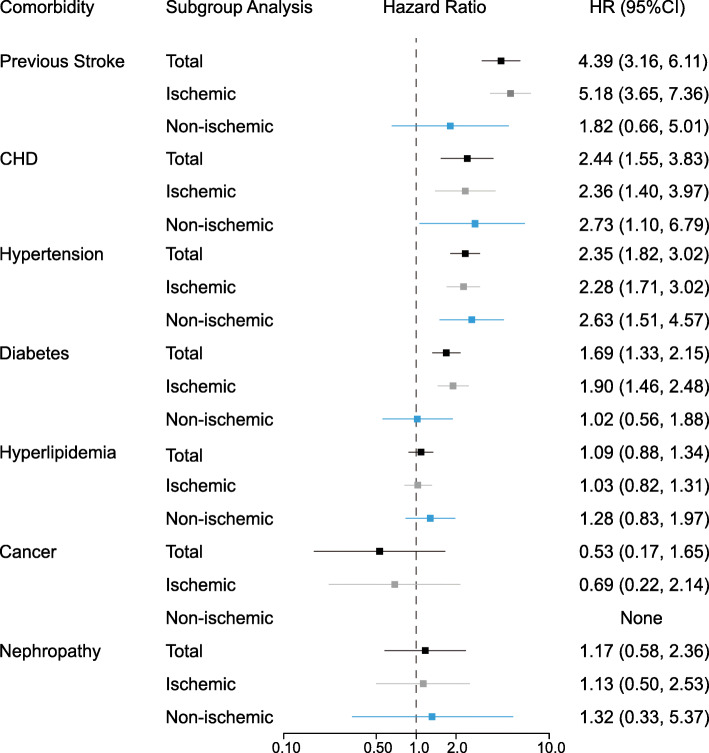
Hazard ratios with 95%CI for 7 pre-existing comorbidities (patients with COPD scarcely had a stroke during the follow-up time) with the risk of stroke, adjusted for sex, age, BMI, current smoking, and drinking. CHD, congenital heart disease

The association between each pre-existing comorbidity and stroke risk are shown in Fig. 1. After full adjustment for age, sex, BMI, current smoking, and current drinking status, the association persisted for four of the eight pre-existing comorbidities, including hypertension, CHD, previous stroke, and diabetes. Furthermore, the four comorbidities were independently associated with both total and ischemic stroke, while CHD and hypertension also had a strong association with non-ischemic stroke.

**Fig. 2:**
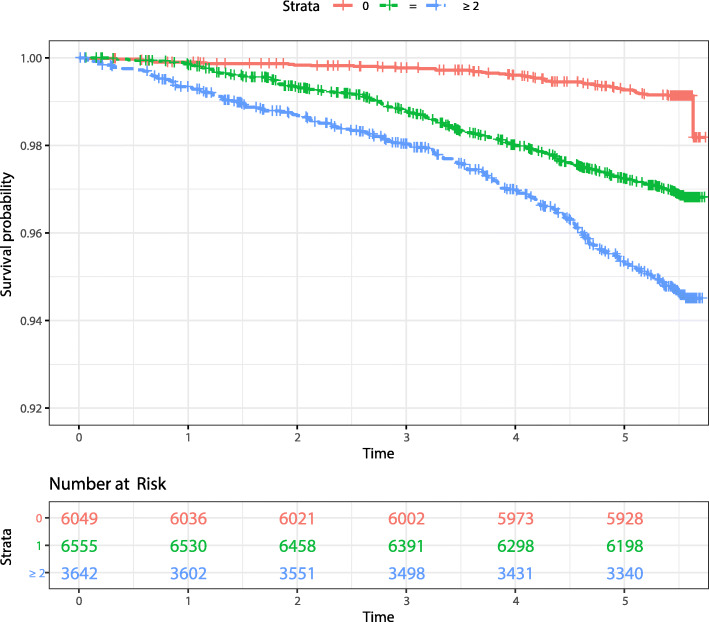
Kaplan-Meier plot depicted the survival proportion of incident total stroke according to groups of total pre-existing comorbidities

A Kaplan-Meier plot shows that the cumulative incidence increased, as the total pre-existing comorbidity changed from 0 to ≥ 2, in a dose-dependent fashion (Fig. 2). As shown in Table 2, we observed that the participants with 1 or ≥ 2 pre-existing comorbidities experienced a significantly increased risk of total stroke in crude models, minimally adjusted models, and models adjusted for sex, age, BMI, smoking, and drinking status (*P* < 0.001), in a dose-response fashion. Compared to participants without pre-existing comorbidities, the multivariable-adjusted HRs for total stroke were 1.96 (95% CI: 1.44–2.67, *P* < 0.001) among those with 1 pre-existing comorbidity, and 2.87 (95% CI: 2.09–3.94, *P* < 0.001) among those with ≥ 2 pre-existing comorbidities. Similar risk associations were observed for both ischemic stroke and non-ischemic stroke.

**Table 2:** Age and multivariable adjusted hazard ratios (95%CI) for incidence of stroke by total pre-existing comorbidities

		Total pre-existing comorbidities						*P* trend
		0		1		≥ 2		
		HR	95%CI	HR	95%CI	HR	95%CI	
Total stroke	Cases/Population	53/6049		203/6555		193/3642		
	Model 1	1.00	Referent	3.60	2.66, 4.87	6.28	4.62, 8.49	< 0.001
	Model 2	1.00	Referent	2.02	1.49, 2.74	3.04	2.24, 4.14	< 0.001
	Model 3	1.00	Referent	1.96	1.44, 2.67	2.87	2.09, 3.94	< 0.001
Ischemic stroke	Cases/Population	42/6049		167/6555		140/3642		
	Model 1	1.00	Referent	3.47	2.47, 4.88	6.31	4.48, 8.87	< 0.001
	Model 2	1.00	Referent	1.93	1.37, 2.72	3.04	2.15, 4.29	< 0.001
	Model 3	1.00	Referent	1.87	1.32, 2.65	2.89	2.03, 4.12	< 0.001
Non-ischemic stroke	Cases/Population	12/6049		49/6555		37/3642		
	Model 1	1.00	Referent	4.10	2.13, 7.90	6.11	3.13, 11.93	< 0.001
	Model 2	1.00	Referent	2.38	1.23, 4.60	3.06	1.56, 6.02	0.001
	Model 3	1.00	Referent	2.30	1.17, 4.47	2.80	1.39, 5.63	0.005

Model 1: unadjusted model; Model 2: adjusted for age and sex; Model 3: further adjusted for BMI, smoking and drinking status

Sensitivity analyses were performed by repeating the primary analyses after excluding the first 1 year of follow-up. Similar risk associations were observed for 1 pre-existing comorbidity [total stroke: 2.15 (1.57, 2.96); ischemic stroke: 2.07 (1.45, 2.96); non-ischemic stroke: 2.46 (1.23, 4.95)] and ≥ 2 pre-existing comorbidities [total stroke: 2.84 (2.03, 3.95); ischemic stroke: 2.83 (1.95, 4.11); non-ischemic stroke: 2.84 (1.36, 5.94)] .

### Interaction between comorbidities and age in association with stroke risk

We further assessed the combined effect of age and pre-existing comorbidities on stroke risk in men and women, separately. As shown in Additional file 1, increased age was associated with a higher risk of stroke across different comorbidity categories. A higher number of pre-existing comorbidities was related to higher stroke risk in both younger and older participants. In particular, the combination of increased age and a higher number of pre-existing comorbidities had the highest risk of total stroke (fully adjusted HR = 23.52 (95% CI: 10.75–51.48); *P* < 0.001 in men and 31.26 (95% CI: 15.10–64.72); *P* < 0.001 in women), compared to younger participants without pre-existing comorbidities. The interaction was in a positive direction on an additive scale. Additionally, women have higher HRs than men in each category, indicating that the interaction had different effects between men and women. Sensitivity analyses were performed after excluding the first year of follow-up, and proved the robustness of findings in the primary analysis.

## Discussion

### Principal findings

In this prospective community-based cohort, pre-existing comorbidities including hypertension, CHD, diabetes, and previous stroke were independent risk factors for total and ischemic stroke. A higher number of pre-existing comorbidities was associated with an increased risk of total stroke and stroke subtypes, in a dose-dependent fashion. Furthermore, there were positive interactions on an additive scale between pre-existing comorbidities and age in association with stroke risk in both men and women.

### Associations of pre-existing comorbidities with stroke risk

Our study demonstrated a positive association between pre-existing comorbidities and stroke risk. After adjusting for age, sex, and other potential confounding factors, previous stroke (compared with those without stroke history) was the strongest risk factor of total stroke, followed by CHD, hypertension, and diabetes. Previous stroke was reported as an independent predictors of long-term survival in stroke patients [33]. Approximately three fourths of strokes in the US are recurrent strokes [34]. After an initial stroke, the risk of recurrence or death was also high among the Chinese population, with more than 40% experiencing a recurrent stroke within 5 years [35]. The association in our study between stroke with hypertension and CHD is in accordance with that previously reported [36–39]. It is believed that cardiovascular risk factors are related to stroke. Hypertensive disorders promote stroke development through increased shear stress, endothelial dysfunction, and large artery stiffness that transmits pulsatile flow to the cerebral microcirculation [40]. In addition, diabetes was an independent risk factor for stroke, which may be due to the dysfunction of cerebral microvasculature caused by hyperglycemia and insulin resistance [41]. Subgroup analysis showed that only ischemic stroke was associated with diabetes, probably due to differing mechanisms in the pathogenesis of stroke subtypes. Results from the Asia Pacific Cohort Studies Collaboration showed that in both Asian and non-Asian populations in the Asia-Pacific region, total cholesterol was strongly associated with the risk of ischaemic stroke [42]. However, we did not observe a significant association between hyperlipidemia and any kind of stroke.

More importantly, our results show that the risk of stroke increased significantly with the higher number of pre-existing comorbidities, after adjustment for other potential confounding factors, and the risk associations were similar for various subgroups. Participants with ≥ 2 pre-existing comorbidities had a nearly three-fold greater risk of stroke compared with those without pre-existing comorbidities. This is in accordance with a case-control study in Swedish [43], which indicated that the risk of stroke in young adults increased with the number of diagnosed comorbidities.

Notably, consistent with a previous report [5], we also found that compared with younger participants without comorbidities, those with ≥ 2 pre-existing comorbidities in the elderly group exhibited the highest risk of stroke, suggesting that the accumulation of cardiovascular risk factors significantly increased the occurrence of stroke, especially in elderly groups. The combination of pre-existing comorbidities and age showed a drastic synergistic effect leading to stoke. There are important public health implications based on our findings. Special attention should be given to elderly patients with a higher number of pre-existing comorbidities for stroke prevention. For patients with a specific pre-existing comorbidity, consulting a primary care provider about the risk of stroke will be helpful to assure that patients accurately perceive their risk and understand how to manage these risk factors.

### Strengths and limitations

Although efforts have been devoted to decipher the association between a single comorbidity and stroke risk [36–42], in this study, we summarized the impact of eight individual pre-existing comorbidities and the number of pre-existing comorbidities on stroke risk in the general population. The deliberate and prospective design, large sample size, and complete and follow-up further ensure the reliability of our findings. However, our study has several limitations. First, among non-ischemic stroke category, a large percentage of undetermined stroke is likely to be undiagnosed ischemic stroke, which may lead to the dilution bias in the results. Second, many comorbidities, such as psychiatric disorders [21–23], gouty arthritis [44], kidney stones [45], and rheumatoid arthritis [46], have been shown to be important factors associated with stroke risk. However, due to the specific study design, mental conditions such as dementia [47] were not included in our study. Additionally, we lack the information regarding liver disease, hemiplegia, AIDS, and peptic ulcer disease, essential to calculating the Charlson Comorbidity Index (CCI), one of the most appropriate measurements of comorbidity [27, 28]. Estimation with CCI will further expand our understanding of the aggregate burden of comorbidity among people at stroke risk. Thirdly, comorbid conditions such as CHD, COPD, previous stroke, and cancer at baseline were collected based on self-reported medical history rather than register-based hospital data, which may lead to misclassification due to the potential social desirability bias and recall bias [48]. Fourthly, residual confounding may exist due to unmearsured risk factors in our study. In addition, given that our study is based on the homogenous Han Chinese population, our results may not be generalizable to other ethnic populations. Another limitation is that the adjusted HRs estimated in the stratification analysis (Additional file 1) might be inaccurate due to the limited cases in individual groups. Finally, information for patients with severe cancer, severe disability and severe psychiatric disturbance was not included due to the difficulty in commuting, communication, and physical examination, and we thus have no such data available for further analysis.

## Conclusions

We observed that the number of pre-existing comorbidities was independently and positively associated with increased risk of ischemic and non-ischemic stroke in the community-dwelling population. Furthermore, older participants with a higher number of pre-existing comorbidities experienced the highest risk of stroke, both in men and women. Our findings suggest that public health and clinical efforts may be beneficial to communicating stroke awareness and prevention messages to populations with a higher number of pre-existing comorbidities, particularly the elderly. Providing personalized information concerning stroke risk for patients with specific pre-existing comorbidities may be an effective approach to prevent and manage stroke events in the corresponding populations.

## Supplementary Information

**Additional file 1.**Combined effects of total pre-existing comorbidities and age on stroke risk in men and women respectively.

## Data Availability

The datasets generated and/or analyzed during the current study are not publicly available due to the restrictions of containing information that could compromise the privacy of research participants, and are available from the corresponding author Shujun Gu on reasonable request.

## Abbreviations

CHD: Congenital heart disease
COPD: Chronic obstructive pulmonary disease
TC: Total cholesterol
TG: Triglyceride
HDL-C: High density lipoprotein cholesterol
LDL-C: Low density lipoprotein cholesterol
BMI: Body mass index
HR: Hazard ratio
CI: Confidence intervals
RERI: Relative excess risk due to interaction
CCI: Charlson comorbidity index
