# Supplementary material for: Association of total pre-existing comorbidities with stroke risk: a large-scale community-based cohort study from China
Source: BMC Public Health. 2021 Oct 21;21:1910. doi: 10.1186/s12889-021-12002-1 (PMC8529731; doi:10.1186/s12889-021-12002-1)
Supplement: Supplementary file 1 — Additional file 1.Combined effects of total pre-existing comorbidities and age on stroke risk in men and women respectively. [file 12889_2021_12002_MOESM1_ESM.pdf]

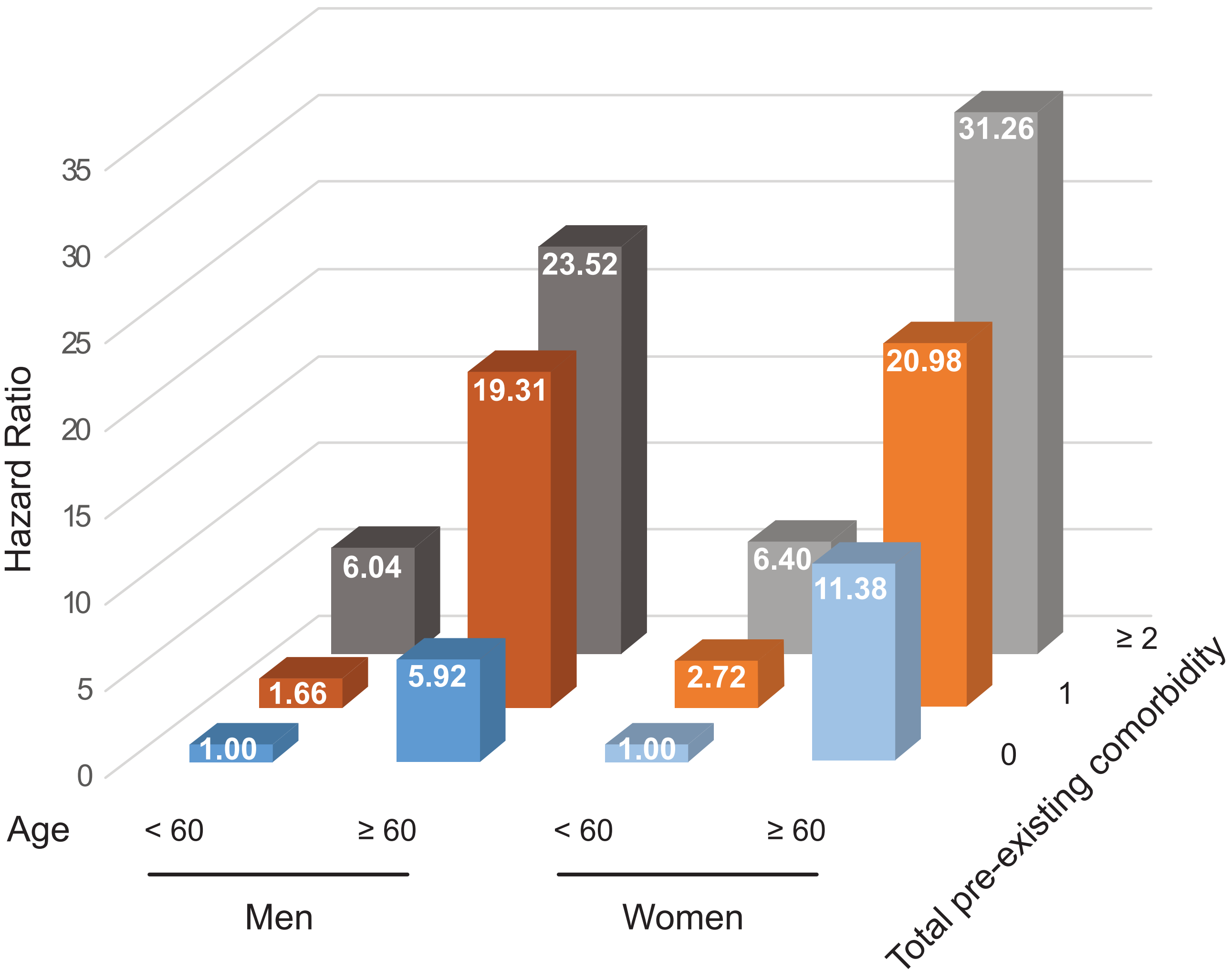

Combined effects of total pre-existing comorbidities and age on stroke risk in men and women respectively. The Cox proportional hazard models were adjusted for BMI, current smoking, and drinking status.
